# Erythema multiforme in a Central American tapir (*Tapirus bairdii*) calf, clinical case report

**DOI:** 10.3389/fvets.2023.1162819

**Published:** 2023-05-17

**Authors:** Luana Hernández Wolters, Alejandro Striedinger Cardona

**Affiliations:** Technical Department, La Aurora National Zoo, Guatemala City, Guatemala

**Keywords:** Perissodactyla, vesicular skin disease of tapirs, glucocorticoids, erythema multiforme, skin disease, antibiotics, drug reaction, amoxicilin

## Abstract

A 7-month-old male Central American tapir with a history of recurrent respiratory diseases and repeated prolonged administration of amoxicillin with clavulanic acid presented with lethargy, severe hyperalgesia, and interscapular ulcerating vesicular lesions with serosanguineous content, as well as dorsal skin peeling, oral ulcers, and thoracic limb, abdominal, and submandibular pustules with surrounding erythema and alopecia. The histopathological analysis and clinical manifestations were compatible with erythema multiforme, which was treated with daily wound cleaning and silver sulfadiazine cream application, as well as oral prednisolone for 15 days, with five daily tapering doses, achieving clinical improvement and an adequate cure of the disease. Vesicular dermatitis syndrome of tapirs is a disease complex including erythema multiforme, a condition rarely diagnosed, since the analyses required for a definitive diagnosis are almost never performed; therefore, it is important to carry out the necessary tests that allow the differentiation of the dermatopathies included in this complex of vesicular skin diseases.

## 1. Introduction

Skin diseases are one of the most common illnesses in tapirs both in the wild and under human care. Vesicular dermatitis syndrome (VDS), the best-known dermatopathy in this species, is a condition with a variable, and in most cases unknown, etiology, and therefore, laboratory assessment and complementary diagnostic tests are needed to confirm a definitive diagnosis (1–3).

Erythema multiforme (EM) is a rare immune-mediated mucocutaneous disorder affecting skin and mucosae that has been previously described in dogs, cats, horses, livestock, and wild animals (4–6). It produces cutaneous inflammation, thereby manifesting as a wide range of lesions, including macules, papules, crusts, prominent target-shaped erythematous lesions, vesicles, bullae, ulcers, hyperkeratotic plaques, and alopecia (7–9).

One of the causes of this disease is thought to be an antigen-induced hypersensitivity reaction mediated by T-cell cytotoxin release, resulting in apoptosis of the affected keratinocytes. This immune response can occur even weeks after the initial stimulus (4, 8, 9). In a study conducted by Scott et al. (10), EM was caused by drugs in 26 out of 44 dogs. Other authors also mention antibiotic, anti-helminthic, and anti-inflammatory drugs, pesticides, vaccines, viruses, and neoplasia as possible triggers of EM (4, 5, 7, 8, 10, 11). Since most cases have an unfavorable prognosis, a prompt histopathological analysis is required for diagnostic confirmation. This test generally shows keratinocytic apoptosis throughout the epidermis and lymphocytic, neutrophilic, and eosinophilic infiltrates, with occasional vasculitis and hair follicle lesions (8, 9).

The purpose of this article is to disclose the clinical manifestations, diagnosis, and treatment of erythema multiforme in a tapir, as well as to explain the relation that this disease and the vesicular dermatitis syndrome of tapirs share.

## 2. Case description

### 2.1. Clinical history

A male Central American tapir (*Tapirus bairdii*) was born under human care at the La Aurora Zoo, located in Guatemala City, and raised by its mother. Both are housed in a 249 m^2^ open-air sand substrate exhibition with areas of sunlight and natural tree shade, a waterfall, and a 32.6 m^2^ pond in which they can swim freely. The enclosure has a 5.62 m^2^ sand substrate sleeping quarter with natural ventilation and infrared light to maintain a stable temperature at night. Their diet consists of chard, bananas, carrots, Mazuri^®^ concentrate (Wild Herbivore Diets), Omalina 300^®^ concentrate, and alfalfa hay, offered twice a day, with fresh water *ad libitum*. The tapir was unvaccinated, but it was recently dewormed with praziquantel and ivermectin.

At approximately 1 month of age, the calf presented with cough, seromucous nasal secretion, stridor, and pulmonary rales and received empirical oral treatment with 30 mg/kg of cephalexin every 8 h for 7 days and 100 mg of acetylcysteine every 24 h for 5 days, after which the cough persisted, but all other manifestations ceased. Two weeks after finishing the antibiotic treatment, the cough continued, and bilateral pulmonary rales were detected, for which 25 mg/kg of oral amoxicillin with clavulanic acid was administered every 8 h for 14 days. Six weeks later, the tapir had new episodes of cough; consequently, chest X-rays were taken, showing pulmonary infiltrates with a bronchial pattern and diffuse pulmonary opacities suggestive of pneumonia. Therefore, he was treated with 25 mg/kg of amoxicillin with clavulanic acid every 8 h for 14 days, 5 mg/kg of acetylcysteine every 12 h for 5 days, 0.064 mg/kg of bromhexine every 24 h for 14 days, and 1 mg/kg of omeprazole every 24 h for 15 days. One month after the end of treatment, the tapir started coughing once more, this time receiving 10 days of oral treatment with 25 mg/kg of amoxicillin with clavulanic acid every 8 h, 3.2 mg/kg of acetylcysteine every 24 h, 0.2 mg/kg of bromhexine every 24 h, and dietary vitamin and mineral supplements.

**Figure 1:**
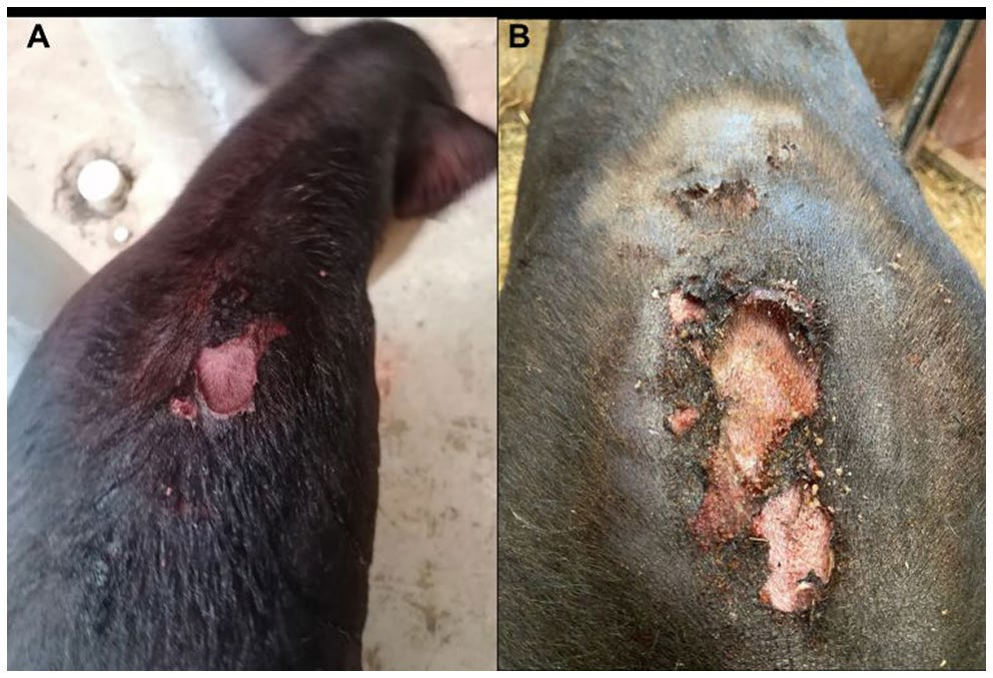
**(A)** Interscapular vesicles and ulcers. **(B)** Ulcers surrounded by shaved skin.

Seven weeks after finishing the last treatment, the tapir, now 7 months old and weighing 110 kg, presented with lethargy, vesicular interscapular lesions with serosanguineous content, and severe hyperalgesia, with physiological constants within range for its species and age. Analgesia with oral phenylbutazone was administered at a dose of 8 mg/kg once daily for 5 days; however, on the same day, the mother began licking the lesions, thus rupturing the vesicles and ulcerating the skin (Figure 1). Superficial sedation with 2 mg/kg of intramuscular xylazine and physical restraint were necessary for blood sampling and wound examination, during which the vesicles ruptured, leaving open wounds with exposed pink tissue without drainage surrounded by thin, friable, swollen skin. The area was shaved, dead tissue was removed, a skin biopsy was taken, and the wounds were cleaned with povidone–iodine and Microdacyn^®^ super-oxidized solution. Subsequently, topical Dermakral^®^ (polymyxin B sulfate, bacitracin zinc, neomycin, and lidocaine hydrochloride) and silver sulfadiazine creams, single doses of intramuscular phenylbutazone at 8 mg/kg, 5 mL of Roborante Calier (calcium phosphorylcholine chloride, casein peptides, and vitamin B12), and 1 mL/ 50 kg of a selenium and vitamin E compound, and subcutaneous ivermectin at 0.2 mg/kg were administered. Finally, 4 mg/kg of intramuscular tolazoline were used for anesthetic reversal, achieving an adequate recovery, after which he was separated from his mother to avoid wound exacerbation.

**Figure 2:**
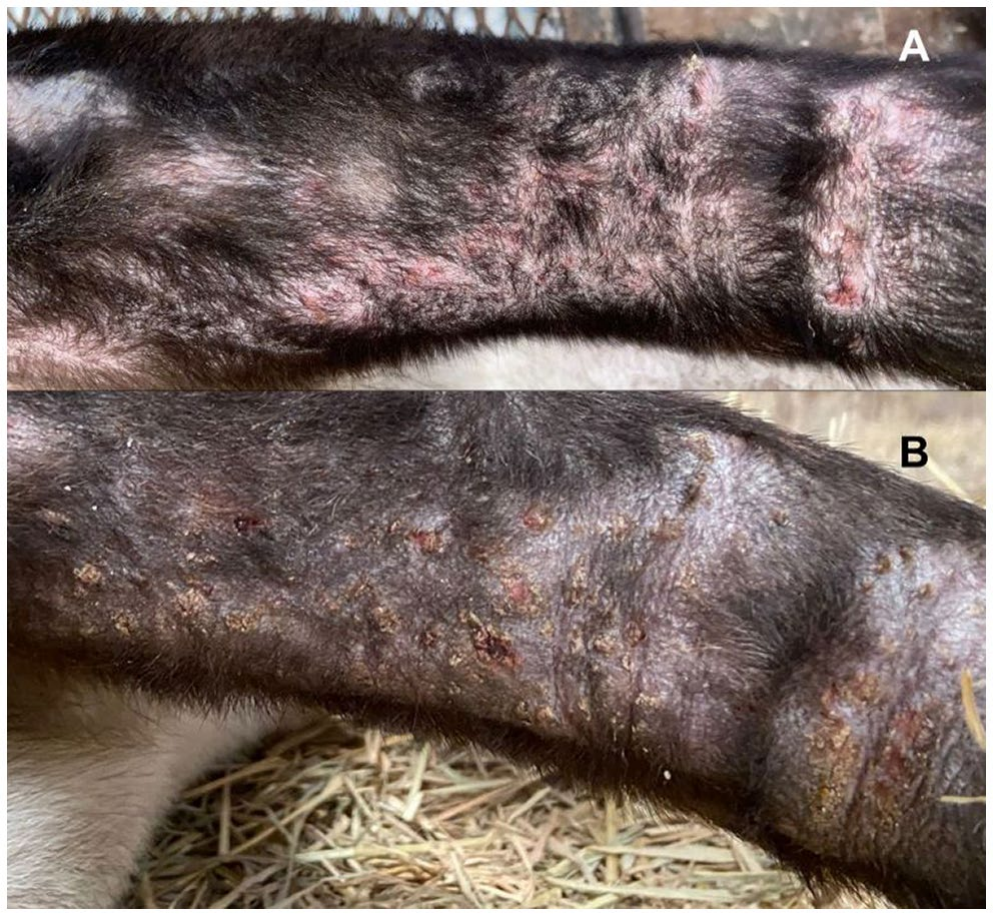
**(A)** Pustules surrounded by alopecic, swollen, and erythematous areas in the caudal region of the right thoracic limb. **(B)** Dried pustules with crusts surrounded by erythema and persistent alopecia in the caudal region of the right thoracic limb.

**Figure 3:**
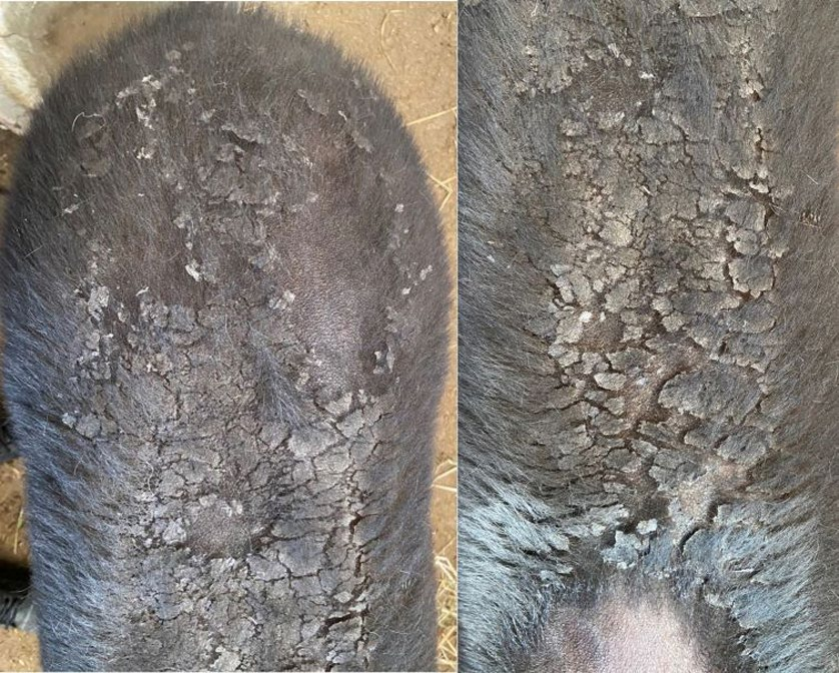
Cracked skin with peeling and detachment in the tapir’s dorsum.

A few days later, pustules surrounded by areas of alopecia, erythema, and swelling appeared in the submandibular and lumbar regions, the dorsal aspect of both thoracic limbs, and the caudal abdominal and inguinal areas (Figures 2A). Cracked skin with peeling and detachment surrounded the lumbar wound. This flaking worsened during the following days, spreading to all of the tapir’s dorsum, from its rump to its head (Figure 3), but the ulcerated areas showed a decrease in hyperalgesia and erythema and signs of wound healing.

### 2.2. Diagnostic assessment

Blood tests were performed during sedation, showing an increase in hematocrit (43.7%; expected range: 25.0–43.0%) and hemoglobin (15.6 g/dL; expected range: 8.0–15.2 g/dL), as well as an increase in blood urea nitrogen (16.8 mg/dl; expected range: 4.1–16 g/dL) and alanine aminotransferase (100 U/L; expected range: 7–30 U/L). One month later, the tests were repeated, and eosinophilia (2.6 ^*^10^3^ cells/μL; expected range: 0.0–0.515 ^*^10^3^ cells/μL) was observed.

A Diff-Quik stain and microscopical analysis of the vesicles’ content was performed, during which was observed an abundance of neutrophils, lymphocytes, and red blood cells, as well as few eosinophils and the absence of bacteria.

**Figure 4:**
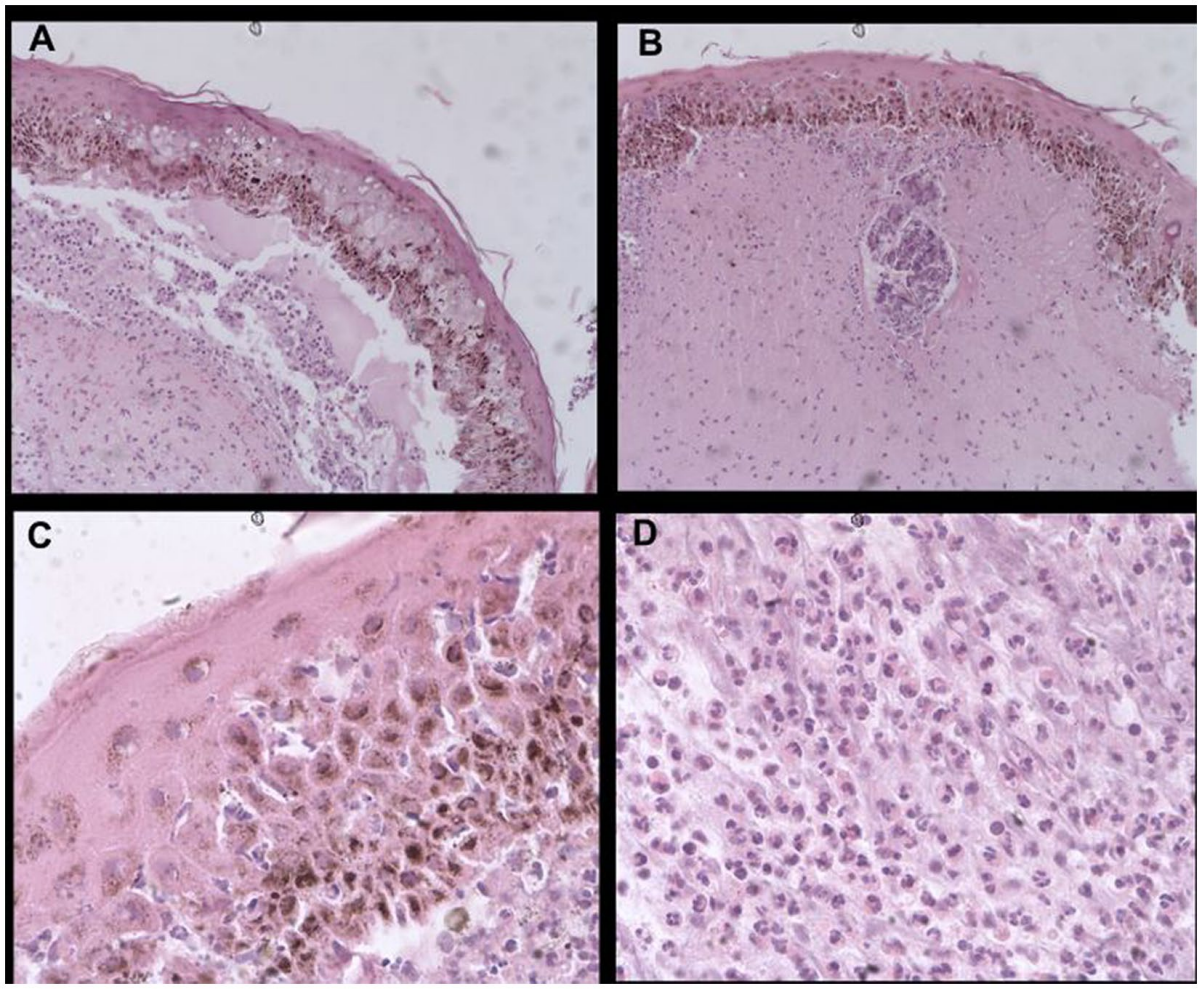
Histological sections stained with hematoxylin–eosin showing: **(A)** widespread epidermal necrosis with flattened cells, dermal detachment, necrotic connective tissue, and diffuse interstitial inflammatory infiltrate in the lower two-thirds of the reticular dermis (40X), **(B)** necrotic hair follicle surrounded with lymphocytic and neutrophilic mural folliculitis, leukocytic interface dermatitis, and mild diffuse interstitial inflammatory infiltrate (40X), **(C)** edema and apoptosis of epidermal keratinocytes embedded in necrotic epidermis with lymphocytic and neutrophilic satellitosis and basal layer hyperpigmentation (100X), and **(D)** reticular dermis with predominantly neutrophilic inflammatory infiltrate (100X).

A skin biopsy was taken with an 8 mm punch on the interscapular skin, including both healthy and ulcerated skin. The histopathological analysis revealed a flattened epidermis with significant hyperpigmentation of the basal layer, a thin laminar stratum corneum, edema, and entrapped keratinocytic apoptosis within areas of epidermal coagulation necrosis, with lymphocytic and neutrophilic satellitosis. In some areas, leukocytic interface dermatitis was present. Cell apoptosis caused detachment of the dermis where necrosis was present in most of the connective tissue, including the blood vessel walls and the hair follicles with lymphocytic and neutrophilic mural folliculitis forming small nodules surrounding the destroyed hair follicles and leaving free hair shafts; the lower two-thirds of the reticular layer showed diffuse interstitial inflammatory infiltrate (Figure 4). According to the characteristics of the lesion found during the histopathological analysis, the authors and dermatopathologist believed these changes were compatible with a progressive form of erythema multiforme.

### 2.3. Treatment and follow-up

Before receiving the skin biopsy result, 100 mg of silymarin and 400 IU of vitamin E were administered orally every 24 h for 1 month, and daily wound cleaning with saline solution was performed, applying silver sulfadiazine cream after each procedure until complete wound healing was attained. After EM was diagnosed, oral prednisolone was added to the treatment for 15 days, tapering the dose every 5 days, starting with 0.5 mg/kg every 12 h, followed by 0.5 mg/kg every 24 h, and finally 0.25 mg/kg every 48 h.

During the initial days of treatment, the pustules on the thoracic limbs started drying, forming crusts with persisting surrounding erythema (Figures 2B); however, the skin peeling spread from the lumbosacral region towards the cervical area, and ulcers were observed in the oral mucosa.

Five days after initiating treatment with prednisolone, generalized skin peeling was observed on the dorsum, with visible healthy skin underlying the detached dead tissue; the pustules found on the thoracic limbs were of smaller size, completely dry, forming crusts, and with reduced surrounding erythema. Daily brushing was performed to remove scale until the skin was completely healed and hair growth was noticed.

After 10 days of treatment, the thoracic limb and abdominal and submandibular regions showed no pustules or crusts, with healthy appearance of all the surrounding tissue, and the dorsal wound was completely healed, without skin peeling and with hair growth in every affected area.

Following the conclusion of the prednisolone treatment, the tapir was active, alert, and responsive, without cutaneous alterations and with natural hair growth on the thoracic limb and lumbar and submandibular regions, which continued until covering all of the injured skin.

## 3. Discussion

Skin diseases are common in tapirs under human care but have also been observed in wild tapirs. Dermatitis in this species may be related to bacterial or viral infections, mycoses, ectoparasites, nutritional deficiencies, poor enclosure hygiene, excessive exposure to sunlight (sunburns), or lack of access to a body of water where they can swim and bathe in Quse and Fernandes (3). VDS is well known in tapirs, and some histopathological analyses have been consistent with those of different diseases, including bullous pemphigoid, dermatitis herpetiformis, the acute hemorrhagic form of staphylococcal hypersensitivity, and erythema multiforme (1, 12).

In canids, the most common cutaneous signs of EM are crust-forming papules, erythematous maculae, and prominent circular plaques; some of these lesions become target-shaped, with erythematous exfoliative concentrical circular areas, and may show ulcers and localized alopecia. These lesions are most commonly located in the axillae, pinnae, oral mucosa, face, and limbs (5, 11). Cats usually present with vesicular, bullous, and ulcerative lesions in the torso and mucous membranes, as well as crusts and extensive alopecic areas (7, 11). Hanley et al. (4) reported one case of a spotted hyena affected by hyperalgesia with erythematous, ulcerative, pruritic lesions that were distributed in the torso, limbs, and perineal and periocular regions. In ferrets, reported manifestations include crusts, erythematous areas, and papules in the inguinal ventral regions, with dissemination towards the axillae and the ventromedial portion of the limbs, as well as hyperkeratosis of the pinnae and digital pads, with thickening and dermal swelling between the digital pads (6). In horses the main lesions consist of non-pruritic, non-alopecic papules and plaques or ring-shaped lesions, with hyperesthesia and loss of skin elasticity, causing secondary lesions such as ulcers and crust formations in the neck and back (13). The cutaneous findings, in this case presented in a tapir, were mostly vesicles with serosanguineous content in the interscapular region, leaving ulcers when ruptured; pustules surrounded by erythema and swelling; alopecia in the ventral aspect of the thoracic limbs and abdomen and in the inguinal and submandibular regions; and skin peeling and detachment in the dorsum and oral ulcers. All of the manifestations observed in the different species show similarities, but even though tapirs and horses are of the same taxonomical order (Perissodactyla), the physiological differences between them are clear and, as a result, cutaneous lesions may vary.

Histopathological analyses in dogs, cats, and ferrets with EM have all revealed keratinocytic apoptosis with lymphocytic satellitosis throughout the epidermis; inflammation with infiltration of lymphocytes and macrophages between the dermis and the epidermis; the additional presence of neutrophils, eosinophils, and plasma cells in ulcerated areas; multifocal intraepidermal micropustules composed of lymphocytes and neutrophils; cellular apoptosis extending to the superficial and infundibular parts of hair follicles and sebaceous glands; and vacuolization of the basal membrane (5–7, 11). In a spotted hyena, in addition to the aforementioned findings, marginalization and extravasation with perivascular accumulation of neutrophils, lymphocytes, and plasma cells have been reported (4). Analyses in horses have also shown epidermal mild-to-moderate spongiosis, hydropic degeneration and necrosis of basal keratinocytes, and pigmentary incontinence of the dermis with superficial edema (13). The main histological manifestations observed in this case were a flattened epidermis with basal layer hyperpigmentation; wide areas of keratinocytic apoptosis and edema entrapped in the necrotic epidermis with lymphocytic and neutrophilic satellitosis; detachment of the dermis with extensive necrosis of the connective tissue, including vascular walls and hair follicles surrounded with lymphocytic and neutrophilic mural folliculitis; and diffuse interstitial infiltrate in the deeper two thirds of the reticular dermis. In more progressive forms of EM, apoptosis can lead to more confluent coagulation necrosis of the epidermis, follicles, adnexa, and dermis. Depending on the stage of the disease and biopsy site sample selection, the presence of more discrete apoptotic cells can be variable. The histologic lesions previously described in EM in different species are compatible with those reported in the present case.

Over time, in both human and veterinary medicine, there had been confusion about the difference between EM, Stevens–Johnson syndrome, and toxic epidermal necrolysis (9). Currently, it is known that these are separate entities; however, erythema multiforme major is still sometimes mistakenly referred to as Stevens–Johnson syndrome. EM can be classified as minor or major depending on the type, extension, and severity of the manifestations. While EM minor consists only of cutaneous lesions, mainly erythematous maculae and papules affecting less than 10% of the body surface, EM major shows severe mucocutaneous lesions, with diagnostic criteria including: presence of vesicles and severe necrotic lesions, involvement of 10-50% of the body surface with any kind of lesion, epidermal detachment of less than 10% of the affected area, and systemic signs such as hyperalgesia, lethargy, pyrexia, and visceral involvement (4, 7–9, 11). In this case, the presence of papules and less than 10% of the body surface being affected were consistent with EM minor, but characteristics of EM major such as mucosal involvement, vesicles, cutaneous necrosis, and severe hyperalgesia were also observed; thus, this case does not fit clearly into either classification.

EM has not been previously described in tapirs, but VDS in tapirs has. Cutaneous signs consisting of papules and vesicles distributed throughout the dorsum, with a tendency in the Central American tapir of extending laterally and causing more severe pruritus, have been reported in these cases of VDS (1, 2, 12). In VDS, lesions tend to begin as fused erythematous papules with serosanguineous content, which release their fluid upon breaking, exposing the dermis and ulcerating the skin. In addition to the cutaneous manifestations, neurological signs are observed in a high percentage of cases, including claudication, ataxia of the pelvic limbs, limb weakness, and episodes of syncope (1, 12). The etiology of VDS is still uncertain (2), although in a survey conducted by Finnegan et al. (1), many tapirs with this disease had a history of intermittent chronic respiratory infections either previous to or during the presence of the cutaneous manifestations, coinciding with this case’s history. Therefore, a wide variety of laboratory tests, including histopathological analysis, are required to obtain a definitive diagnosis (1, 3). Histopathological analyses usually show subepidermal vesicles containing abundant fibrin, neutrophils, and eosinophils, with subepidermal spongiosis and superficial or even deep necrosis; in the viable epithelium, the presence of neutrophils, eosinophils, and erythrocytes can be observed. In the dermis, perivascular edema and severe hemorrhage can be observed, and the collagen layer usually shows degeneration and mild neutrophilic and eosinophilic infiltrates, but skin appendages are rarely affected (1, 12). The histopathological characteristics described in this tapir were similar to those reported in the literature.

The history of constant and prolonged administration of amoxicillin with clavulanic acid coincides with the information reported on humans and canids, on whom this and other beta-lactam antibiotics have been evidenced to cause EM (4, 10). However, there is a risk that other previously administered drugs could be the causal factor of EM in this case.

The treatment for EM requires discontinuing any drug suspected to be causing the pathology (in case of a drug-related etiology); fluid therapy initiation is recommended in cases of widespread skin lesions and antibiotic use to prevent a secondary wound infection (7). Treatment with glucocorticoids or other immunosuppressive drugs is still controversial, since cases have been reported where these drugs led to a successful resolution of the disease (14), particularly in idiopathic cases of EM (8), and where even the prolonged administration of prednisolone did not demonstrate any improvement (6, 7). New treatment methods have been reported in dogs and cats, such as administration of human intravenous immunoglobulins (IVIG) as monotherapy or in addition to immunosuppressive drugs, both of which have shown promising results (7, 14). This case reports an example in which prednisolone therapy in a tapir was effective, as it suppressed the signs of the disease.

## 4. Conclusion

Vesicular dermatitis syndrome of tapirs is a disease complex encompassing erythema multiforme, a rarely diagnosed condition, since the analyses required for a definitive diagnosis are almost never performed; therefore, it is important to carry out the necessary tests that allow the differentiation of the dermatopathies included in this complex of vesicular skin diseases. Even though the histological characteristics of EM are similar in different species, clinical manifestations vary. Further research is needed to determine if antibiotics can be a cause of EM in tapirs. In the same way, the search for more effective treatment methods is recommended.

## Data availability statement

The original contributions presented in the study are included in the article/supplementary material, further inquiries can be directed to the corresponding author.

## Ethics statement

Ethical review and approval was not required for the animal study because No experimentation was carried out during this study. Animal welfare complied with ALPZA and industry standard guidelines. Written informed consent was obtained from the animal’s owner for the publication of this case report.

## Author contributions

LH performed as the doctor in charge of the clinical case, conducting diagnosis, treatment, and follow-up of the pathology, as well as data collection, research, and manuscript writing and editing. All authors contributed to the article and approved the submitted version.

## Funding

This article was supported by the La Aurora Zoo.

## Conflict of interest

The authors declare that the research was conducted in the absence of any commercial or financial relationships that could be construed as a potential conflict of interest.

## Publisher’s note

All claims expressed in this article are solely those of the authors and do not necessarily represent those of their affiliated organizations, or those of the publisher, the editors and the reviewers. Any product that may be evaluated in this article, or claim that may be made by its manufacturer, is not guaranteed or endorsed by the publisher.
